# Editorial: Complex Immune Mediated Pulmonary Disease: How Genetic Data Can Influence Clinical Practice

**DOI:** 10.3389/fmed.2019.00150

**Published:** 2019-07-02

**Authors:** Martin Petrek

**Affiliations:** ^1^Department of Pathological Physiology, Faculty of Medicine and Dentistry, Palacky University Olomouc, Olomouc, Czechia; ^2^Departments of Immunology and Experimental Medicine, University Hospital Olomouc, Olomouc, Czechia

**Keywords:** complex lung disease, genetics, bench to bedside, translational medicine, editorial

Contribution of genetics to our understanding of pathomechanisms of complex pulmonary disease is undoubtable. Genetic Association Studies (GAS) with candidate genes, Genome-Wide Association Studies (GWAS), or Whole Exome Sequencing (WES) have collectively identified a spectrum of susceptibility loci in diseases such as COPD (1), IPF (2), or sarcoidosis (3). While investigators have started to relate the findings obtained using sophisticated technologies to distinct phases of the disease course or to specific disease phenotypes (4), there is still a gap between research observations and their application to patient management. In this context, the aim of this Research Topic was to provide readers with contributions from translational genetics of complex, immune-mediated respiratory disease, and to explore areas for further research in this field.

Three review and three original articles were accepted by the editorial team (Dr. van Moorsel, Dr. Rivera and the author of this editorial) after passing interactive review with reviewers' names openly disclosed. The collection of these six contributions does not address solely the assessment of genetic background of given lung diseases but also determination of the etiologic factors using genetic methodologies or identification of new biomarkers. In wider sense, the articles in the collection cover also the aspects how to analyse, interpret, and utilize vast amount of data generated by experimental approaches and how to find ways for their practical utilization in clinical decision making.

The first review by Newton et al. ideally fitting within the scope of this Research Topic, presents a concise overview of the state of the art in the application of genetics into the diagnosis and management of interstitial lung diseases. The authors do not focus only on idiopathic pulmonary fibrosis (IPF), a debilitating disease in which a number of gene variants was recently nominated and started to be investigated for their clinical relevance. Further, they discuss yet another disease with distinct immune features, hypersensitivity pneumonitis, in which the role of genetic factors is far from clarified and thus should be investigated (5). In their perspective, the authors suggest application of genetic testing for patient risk stratification including assessing predictive response toward the drugs currently used in patient management or only being tested for their future potential application. The review represents a solid and scholarly introduction for any clinical fellow interested in this area. At the same time, it could provide interested scientists and laboratorians with the relevant clinical context.

The second review by Harishankar et al. characterizes the recent progress in the contribution of genetic variation to susceptibility/resistance to tuberculosis (TB). Besides the role of the most polymorphic genetic system in humans - HLA, the authors list other TB-associated genes with signaling, enzymatic and other (i.e., transcription factor) functions, e.g., Forkhead BoxP1 (FOXP1), C-terminal domain phosphatase, etc. The review emphasizes the importance of population (ethnicity) aspect of these associations with main focus on Asia.

The author of this editorial himself has co-authored a minireview (Kishore and Petrek). Here, the advent of a new technology of massive parallel sequencing (NGS, next generation sequencing) and its application to determination of extensive HLA polymorphism is described in context of investigations of another complex pulmonary disease with immune features, sarcoidosis. It is proposed that fine characterization of HLA at the allelic level will provide precise assessment of existing and identification of novel associations between HLA and sarcoidosis/its clinical phenotypes; the authors also discuss population aspects of sarcoidosis immunogenetic research.

There are also three original papers in this collection. The first one, from van Moorsel lab, shows possible implications of studies of genetic variation in the TERT gene, which affects telomere length, on clinical decision making. Using previously published GWAS/GAS data, Snetselaar et al. performed a metaanalysis of rs2736100 C/A in cancerous and non-malignant disease. They report that while C allele of the investigated SNP positively associates with cancer, including lung cancer, it has a negative association with non-cancerous diseases such as pulmonary fibrosis. Thus, variation in this single gene may be partly responsible for the ambiguous role of telomerase maintenance in disease. In this context, the authors issue a warning of caution regarding usage of telomere maintenance therapies.

In the second original paper of this Research Topic, Baos et al. focus on non-allergic asthma (NA). In extension of their previous work, which characterized possible candidates for NA biomarkers by transcriptomic (mRNA) analysis, the authors have evaluated protein levels of seven nominated genes. Based on the current data including ROC analyses, Chitinase-3-like protein 1 (CHI3L1), elastase-specific protease inhibitor PI3, and namely their combination is proposed as plausible biomarkers of non-allergic asthma itself and possibly also of its severity. This paper may represent translation of gene expression studies to clinical practice.

The last original paper by a joint German and Armenian author team (Hopp et al.) approaches common acquired pneumonia (CAP), namely its septic complication, from a perspective angle of bioinformatics. Analysis of patients' blood transcriptome showed that disease severity was associated with immune suppression due to disturbance in a number of functional immune components and functional processes such as T-cell exhaustion, receptor deactivation or metabolic conversion. The authors also demonstrate epigenetic alterations including chromatin remodeling and related methylation changes. Based on their data, they propose a novel stratification for CAP with possible impact on prognostication.

The review and original articles in this collection do not and cannot address all issues pertinent to area of genetic applications to complex immune mediated lung disease. This area has been under constant progress as evidenced e.g., by most recent reports on new genetic signals for lung function in COPD (6) or current lung-disease oriented applications of EWAS (Epigenome-Wide Association Studies) (7) and PheWAS (Phenome-Wide Association Studies) (8, 9). Thus, this Research Topic can represent only a partial reflection—a window into a given time period of ongoing developments. Despite these limitations, it attracted original and thoughtful contributions and the editorial team believes that these will continue to find their audience, as it could be indirectly indicated by the number of views and/or downloads currently averaging more than 2,000 per contribution.

## Author Contributions

The author confirms being the sole contributor of this work and has approved it for publication.

### Conflict of Interest Statement

The author declares that the research was conducted in the absence of any commercial or financial relationships that could be construed as a potential conflict of interest.
